# Comparison of performance of various leg-kicking techniques in fin swimming in terms of achieving the different goals of underwater activities

**DOI:** 10.1371/journal.pone.0236504

**Published:** 2020-08-03

**Authors:** Marek Rejman, Piotr Siemontowski, Adam Siemienski

**Affiliations:** 1 Department of Swimming, University School of Physical Education in Wroclaw, Wroclaw, Poland; 2 Department of Technology in Underwater Activities, Polish Naval Academy, Gdynia, Poland; 3 Department of Biomechanics, University School of Physical Education in Wroclaw, Wroclaw, Poland; University of Pittsburgh, UNITED STATES

## Abstract

The aim of this study was to compare underwater fin swimming performance using dolphin, flutter and breaststroke kicks with and without diving gear. Performance was evaluated in terms of average swimming velocity. The parameters of spatiotemporal structure of the stroke reflecting to the swimming economy were employed. Conscious modifications in propulsion technique were considered here with the aim of controlling swimming performance. A total of ten professional scuba divers swam at maximal speed underwater for 50m using each of three techniques: dolphin, flutter, or breaststroke kicks. Swimmers’ performance was compared between holding their breath and using breathing apparatus. Two cameras recorded their movements in sagittal and transverse planes. The average swimming velocity (v_av_), stroke length (SL), stroke rate (SR), index of variation of intracycle velocity (VIV_Index_) and stroke index (SI) were estimated. Relative to the other techniques, the dolphin kick without a diving gear demonstrated the highest v_av_ and low SI and VIV_Index_ values, which reflects the most advantageous economy of propulsion at given velocity. Given the lack of statistical differences, using the breaststroke kick and flutter kick when swimming with a diving gear seems to be comparable to dolphin-kick in terms of average velocity and parameters reflecting the economy of propulsion. Thus, a search for fin swimming techniques with the aim of achieving specific goals seemed reasonable. The results suggest, that performance achieved while using various fin swimming techniques was probably controlled by different strategies of leg movements. These strategies revealed differences in a spatiotemporal (SR-SL) structure of the stroke and they were closely associated in terms of the velocity variation decrease.

## Introduction

Fin swimming is a widely practiced aquatic activity. The large surface of the fin facilitates propulsion in competitive fin swimming and underwater hockey. Fins are also used in recreational swimming and scuba diving. They often constitute part of the personal equipment of lifeguards and are basic equipment for industrial and military divers. Various goals are implemented within a broad spectrum of these activities. In competitive fin swimming, the specific skills and goals imply maximizing performance and rational energy expenditure, allowing maintenance it at stable levels over the entire distance [1]. Lifeguards need to approach a victim as quickly as they can, but they also need to economize the efforts in order to achieve efficient towing and perform other rescue actions later on [2]. In scuba diving, it is the diver’s breathing comfort that is the most important. Advanced scuba divers should concentrate on minimizing the consumption of air they breathe, in order to extend the diving time. Divers performing special tasks are required to carry out conscious modifications of the kicking technique according to the changing conditions. In this context, we postulate that any assessment of the performance of kicking techniques in underwater swimming should be made in terms of the specific goals that the swimmers want to achieve. Moreover, we propose that the swimmers' own preferences, based on their specific skills, should be also taken into consideration. Therefore, the traditional understanding of fin swimming techniques as an efficient and economical utilization of the fin surface in order to achieve best performance [3] will be considered in a wide and not merely mechanical context. The quite miscellaneous goals of fin swimming inspired us to formulate a key assumption of this study, namely that conscious using of each kicking technique, reflected by swimming speed and economy of propulsion, might serve to control the optimal (goal-oriented) performance. In this context, fin swimming performance will be assessed through the prism of praxeology, that is, the science of the action-oriented implementation of a goal [4]. Implementation of praxeology as a tool for functional evaluation of sporting activity was successfully adopted [5,6].

Average swimming velocity (v_av_) is the best measure of swimming performance. However, its significance is more pronounced while swimming at a short distance than at a longer one [7] or when swimming holding the breath [8]. Therefore, the economies of fin swimming have been investigated for a long time [9] seeking to quantify it on the basis of fin swimming in connection with the energy expenditure [10–12] and in terms of the fins’ size and stiffness [7–12]. Indeed, the economy of flutter and dolphin kicks with fins have been researched by Zamparo et al. [13,14]. The stroke index (SI) supporting the assessment of one’s ability to maintain high swimming velocity using a small number of strokes, can also be used (imposing some limitations [15]) as overall swimming economy estimation [16] and a determining factor of swimming performance using different strokes at various distances [17]. SI has never been employed in the analyses of fin swimming techniques. Therefore, it is interesting to research the role which this parameter plays in underwater fin swimming performance.

Increases or decreases in v_av_ are determined by combined increases or decreases in SR and SL, respectively [17,18]. Therefore in assessing swimming performance, the stroke length (SL) and the stroke rate (SR) are useful measurement tools from the physiology and biomechanics point of view [7]. Consciously controlling the strategy of the stroke parameters (SL and SR) distribution aids in achieving a high level of technical skill in swimming. This way the competitive swimmers are able to increase the economy of propulsion, reduce the energy cost and the symptoms of fatigue during performance [19]. Such an outcome of control has also been observed in monofin swimming [20, 21]. Nicolas and Bideau [22] reported that optimal SL and SR for different fin designs facilitate the swimming performance. These considerations justify the use of a combination of stroke parameters (SL and SR) to assess fin swimming performance.

Swimming propulsion represents a combination of trunk, arm and leg movements that result in non-uniform body movements and intracycle velocity variation [7]. Assessments of intracycle velocity variation in different competitive swimming techniques have been reported in numerous studies, especially focusing on the relationship between velocity variation and energy cost [23–26]. Barbosa et al. [7] reported that the positive relationship between intracycle velocity variation and average swimming velocity may be frequent in shorter events, while not present in longer events. Rejman et al. [2] undertook a sequential analysis of leg and monofin movements in relation to horizontal velocity variations. Moreover, the aforementioned authors [20] examined the relationship between forces bending the monofin and the angular parameters describing this bending in relation to intracycle swimming velocity. Although changes in intracycle velocity during fin swimming have not been studied as thoroughly as in traditional swimming, it can be assumed that they constitute a tool for assessing fin swimming performance.

It should be emphasised that the average swimming velocity interplays with stroke index, stroke parameters (SR and SL) and intracycle velocity variation. Thus these parameters can be used to assess underwater fin swimming performance. Furthermore, few fin swimming performance studies focusing on the use of different kicking techniques have been published [2,13,14]. No research has investigated propulsive movements using the breaststroke kick with fins.

In response, the aim of this study was to compare underwater fin swimming performance using dolphin, flutter and breaststroke kicks with and without diving gear. Performance was evaluated in terms of average swimming velocity, employing the parameters of spatiotemporal structure of the stroke and reflecting the economy of propulsion. It follows the quest for conscious modifications in fin swimming technique and for controlling the strategy of propulsion generation in order to achieve optimal performance—oriented on a particular goal.

## Materials and methods

### Participants

This study included ten male professional military scuba divers of similar ages (mean = 24.2, SD = 0.92 years) and body compositions (body height—mean = 181.8, SD = 4.29 cm; body mass–mean = 83.9, SD = 4.48 kg). They all possessed the same certificate from the military training itemized procedures. It was thus assumed that they presented a similarly high level of diving proficiency.

The study was reviewed and approved by the Institutional Review Board—Ethical Committee at the University School of Physical Education in Wroclaw, Poland (reference number 023/2017) before the study began. This way, all procedures involving human participants in this study were performed in accordance with the 1964 Declaration of Helsinki and its later amendments or comparable ethical standards. Every participant provided his written informed consent prior to take part in this study.

### Experimental design and tasks

In the first trial, in a random order, divers completed two sets of tasks in a 50-metre pool. The first task was to swim underwater while holding their breath over a 50-metre distance using each of the three techniques: dolphin kick, flutter kick and breaststroke kick with fins. All the participants were able to perform the correct breaststroke-kick with fins—simultaneous, on the same horizontal plane, with dorsal feet flexion during propulsive phase of the stroke. They were asked to swim at maximal speed in a natural prone position. No restrictions concerning the positions of the arms were formulated. Participants voluntarily kept their arms extended over their heads in all of the three kicking techniques, therefore the arm position did not affect the study results. This task was then repeated with participants wearing diving gear (all divers using the same buoyancy jacket, regulator and air tank). The same standard fins with a high level of stiffness (0.65m by 0.25m, with an adjustable open heel-type to ensure suitability for all participants) were used in both tasks. The divers’ standard personal equipment of mask, snorkel and wetsuit differed. The jacket was inflated in order to find each diver’s neutral buoyancy. Between each trial, it was ensured that each participant’s heart rate had returned to pre-exercise levels, before proceeding further. The heart rate was subject palpated at the neck (carotid artery). All the participants familiarized themselves with all the experimental procedures during the warm-up session. Professional military scuba divers are used to performing more complex and challenging tasks on a daily basis, therefore, it is unlikely that the difficulty of the procedures affected the outcome of the research.

**Fig 1:**
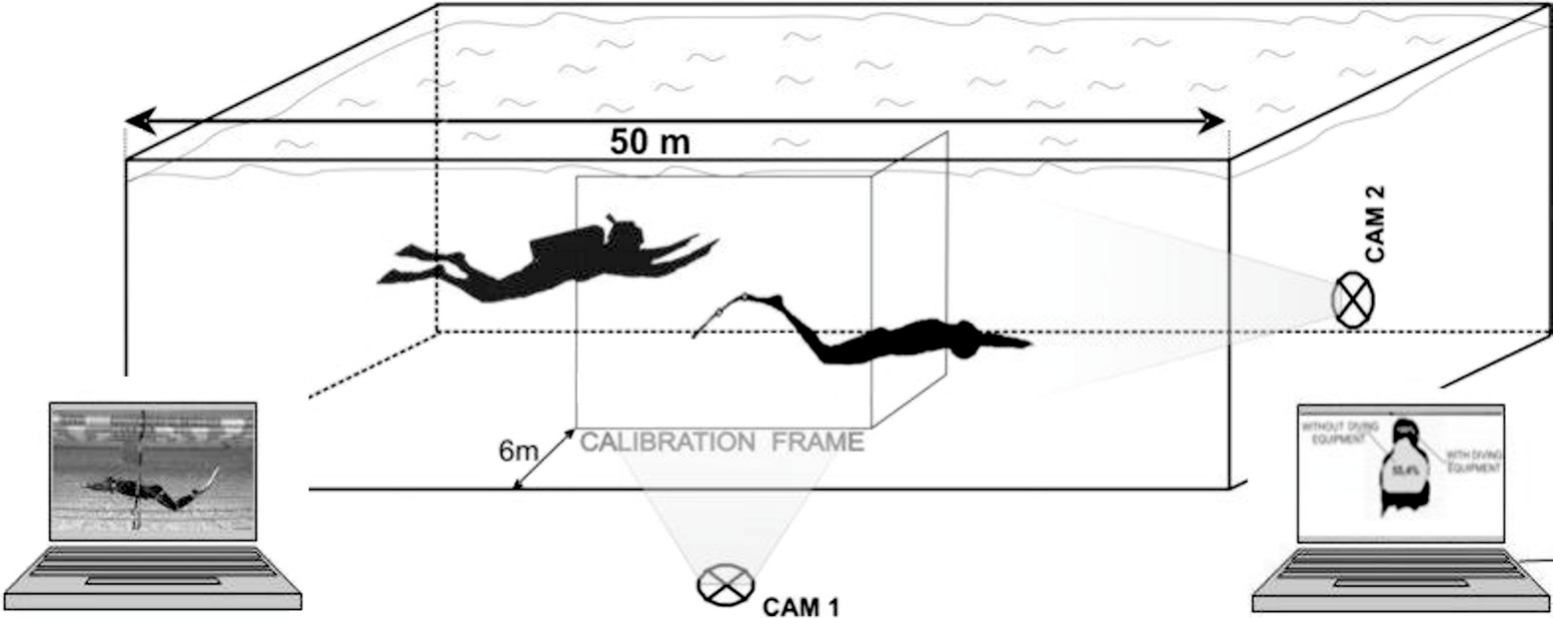
Experimental set-up and data collection.

Two digital cameras (DCR-TRV 22E, Sony, Japan) were used to film all of the divers underwater (Fig 1) at a sampling frequency of 50 Hz. Camera 1 (Cam 1) was located in a stable position near the centre of the pool in order to record the divers’ movements in the sagittal plane. The calibration frame was treated as a reference system. Markers to track the displacement of the hip joint were placed on the swimmers’ bodies [27]. The markers were also placed in the centre of mass of the air tank in order to track the displacement of any additional mass carried by the diver. The raw data were fed into SIMI Motion software (SIMI Reality Motion Systems GmbH, Germany) and analyzed in order to estimate the swimmers’ horizontal average swimming velocity—(v_av_)) and the mean values of the stroke parameters: SL, defined as the distance a swimmer covers in one stroke [m]; and SR, defined as the average number of strokes per time unit [1/s]. Next, the SI was calculated (Eq 1) according to Costill et al. [16]:

$$SI=SL\times v_{av}$$ (1)

where *SI* is the stroke index, *SL* is the stroke length and *v*_*av*_ is the average horizontal velocity.

**Fig 2:**
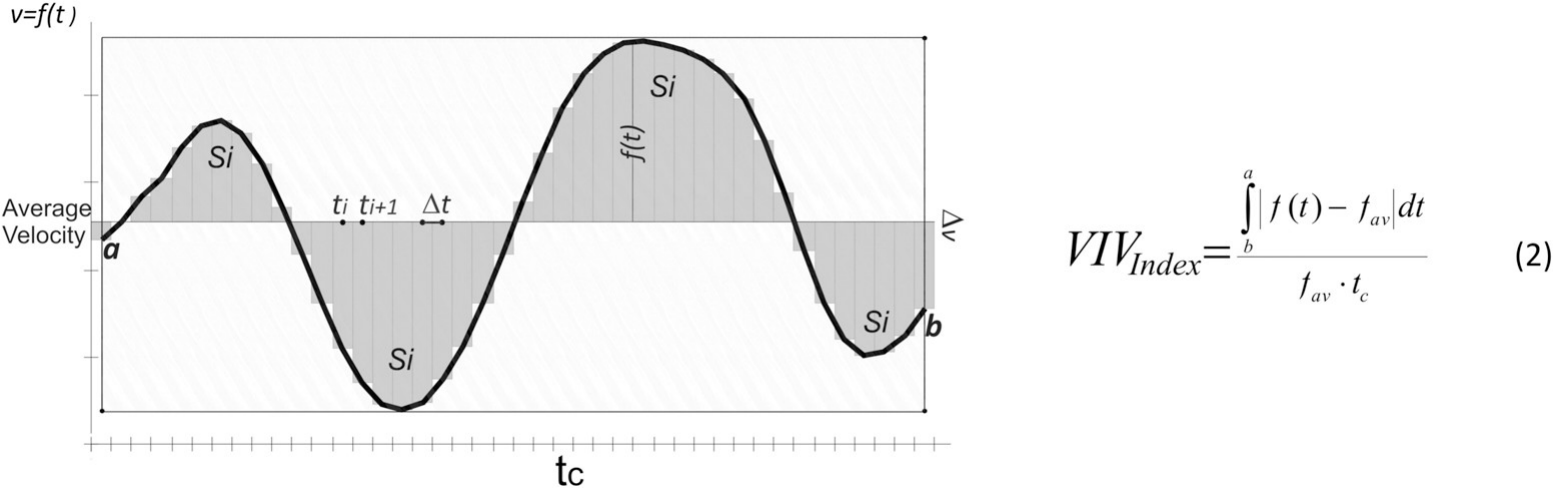
Graphic representation of the definition of the index of variation of intracycle velocity (VIV_Index_). *f(t)*–Instantaneous swimming velocity (*v)* at the point in time (*t*); *f*_*av*_*−*Average horizontal swimming velocity as the function of stroke time (*t*_*c*_*); t*_*i*_*−*Time recorded at each sampling point; *a* and *b*–The points limiting the stroke time (*t*_*c*._*)*.

The index of variation of intracycle velocity (VIV_Index_) was also estimated (Fig 2). This indicator, designed as a dimensionless measure of relative dispersion in the intracycle swimming velocity, describes the ability to minimize the negative drag that is experienced on the swimmers body as a result of its instantaneous accelerations. Here, it was defined as the area enclosed between the curve of instantaneous swimming velocity and the line corresponding to the average velocity, divided by the distance covered, as shown in Equation 2.

Measuring swimming speed is considered a simple way for assessing the performance [7]. Therefore the average swimming velocity (v_av_) was used in this study as one of the main contributing factors, which determines performance. As swimming speed increases, the hydrodynamic resistance that the swimmer must overcome also rises, thus creating an increase in energy cost. Thus, the economy of propulsion can be assessed at given swimming velocity [7]. In this context, when SI decreases from longer to shorter events [28], in accordance with the criterion of velocity maximization with the lowest number of strokes [16], SI can be treated as a reflection of the economization of propulsion generation. Moreover, high swimming velocity is positively associated with intracycle velocity in shorter events, while negative ones in longer events [7] Thus the VIV_Index_, which represents the criterion for minimization of drag by means of avoiding changes in intracycle velocity, can be perceived as a parameter revealing of the economization of propulsion utilization. Increases or decreases in swimming velocity are determined by combined increases or decreases in SF and SL, respectively [15,17,18]. These relationships were treated many times as a tool of assessment of fin swimming performance [13–14, 20–22].

The displacement of the hip of each swimmer was also tracked in one swimming stroke in the following manner: the beginning and the end of the stroke were carefully estimated during a digital motion analysis mentioned earlier. In the dolphin-kick—from the highest heel position before downward phase, until the highest heel position after the upward phase; in the flutter-kick—in the same way as in the dolphin kick, but with one (more visible) leg; for the breaststroke—from the beginning of the initial phase (the first motion after straightening of the legs), through push-off until the end of the gliding phase with the legs straight (beginning of the next movement).

The center of mass of the air tank was also estimated experimentally (on land). The fulcrum in which the horizontally positioned tank remained in equilibrium was found. The projection of this point was transferred onto the tank body (as a line). Then, at this line the half the transverse dimension of the air tank was estimated with a caliper. In this point the center of mass was marked. This marker was visible when the air tank was inside the buoyancy jacket.

**Fig 3:**
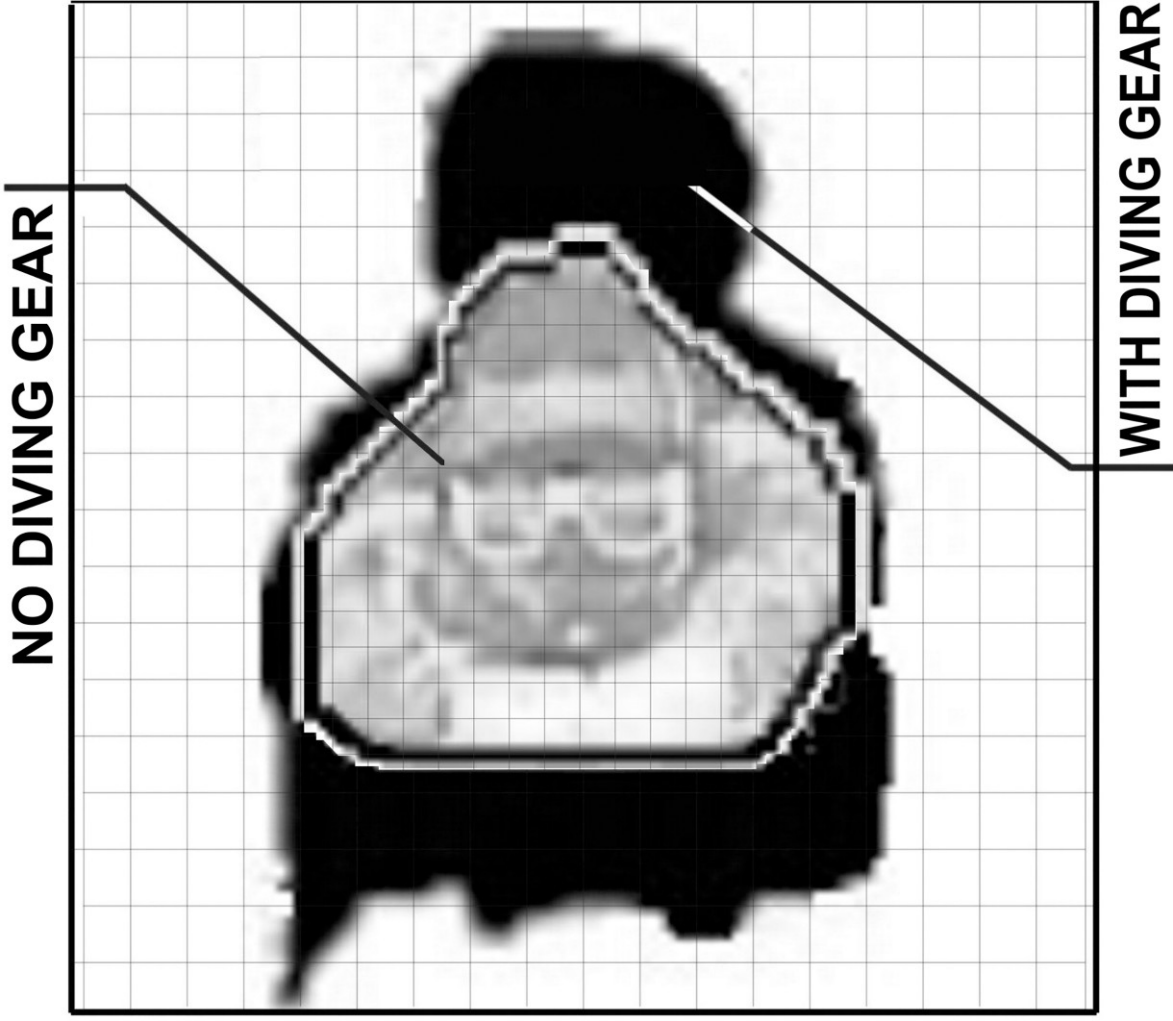
Estimation of cross-sectional area for scuba divers without and with (black) diving gear.

The estimated cross-sectional areas of the divers’ bodies when swimming with or without diving gear were compared (Fig 3). This estimation was based on a single snapshot of each diver’s body, selected from images recorded in the transverse (front) plane by the second underwater camera (Cam 2, Fig 1). All snapshots were taken when the marker located on the swimmers’ hips, visible on Cam. 1, came through the second calibration frame, joined at a right angle to the aforementioned frame (Cam. 1 and Cam 2 were synchronized) (Fig 1). Using Corel Photo Paint software (Corel Corp., Canada), these images were then enlarged and spread out to pixel-level scale. The total sum of visible pixels bordering the area of the diver’s body was used to calculate the diver’s cross-sectional area (the area of projection of the legs was not taken into consideration owing to the differences in the kicking techniques analyzed). Normalization was performed against the cross-sectional area.

### Statistical analysis

Statistical analyses were performed using Statistica 12.0 software (StatSoft, USA). The raw statistical analysis of the described parameters facilitated 3x2 analysis of variance for dependent samples (ANOVA for symmetrical factorial design). The variables were introduced as two groups of qualitative predictors: propulsion generation techniques (dolphin kick, flutter kick and breaststroke kick) and diving gear (swimming with and without equipment). The results obtained (the average margin) were normalized. The statistical significance of each difference was estimated by Duncan’s post-hoc test, thus creating a foundation for research on the dependencies between all of the parameters (Spearman’s rank correlation).

## Results

**Fig 4:**
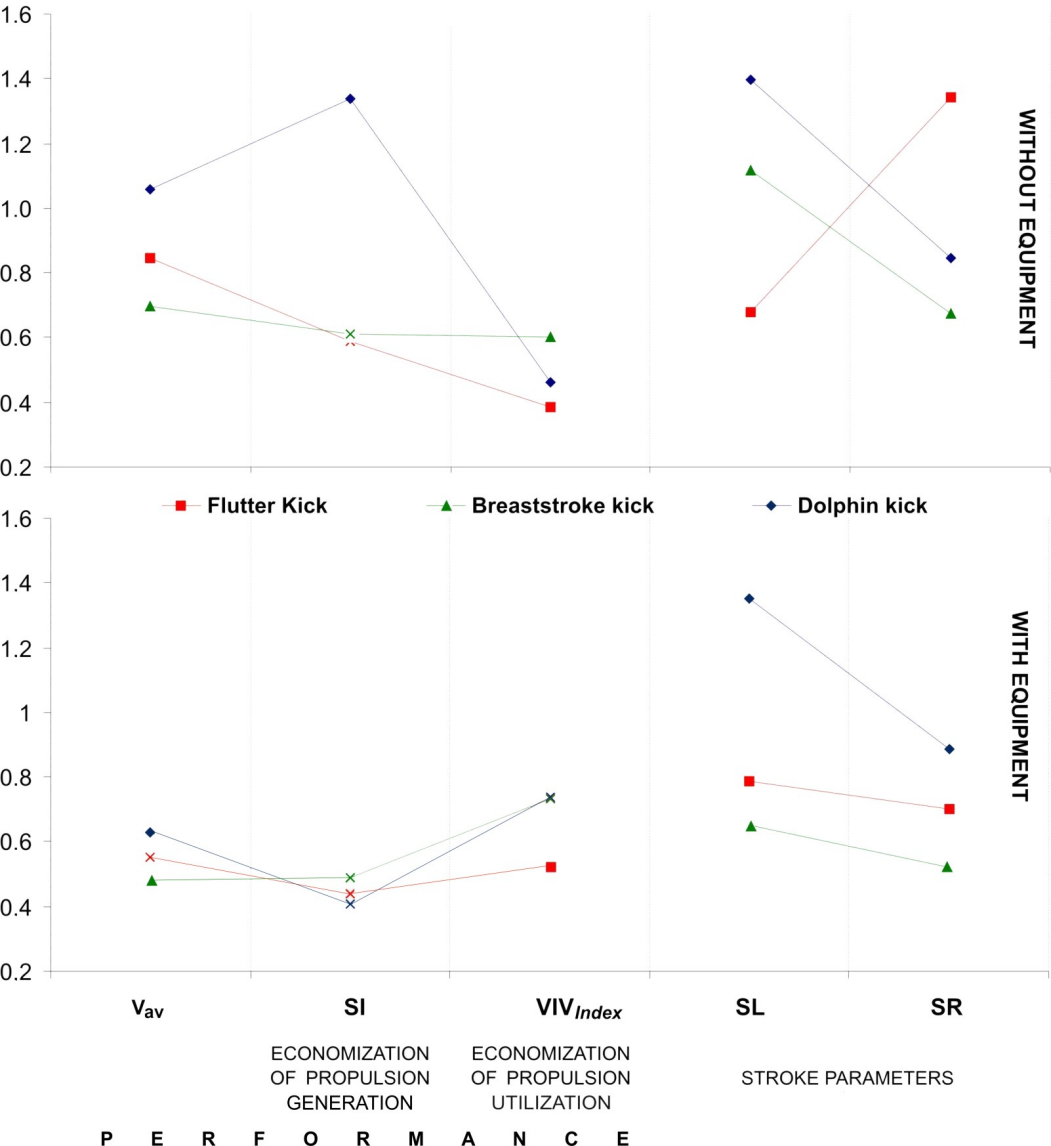
Graphs of marginal mean values of the studied parameters for dolphin kick, flutter kick and breaststroke kick with and without diving gear (Parameters that were not statistically different are marked with a cross). v_av_−Average swimming velocity; SI–Stroke index; VIV_Index_−Index of variation of intracycle velocity; SL–Stroke length; SR–Stroke rate.

As shown in Fig 4, the dolphin kick presented the fastest (v_av_) swimming technique in both trials. In addition, the stroke index (SI) strongly differentiated this technique from the others in swimming without diving gear (Table 1). Moreover, only for the flutter kick with diving gear did the index of variation of intracycle velocity (VIV_Index_) significantly differ from the other techniques.

**Table 1:** Statistical significance of differences (p-values) in analyzed parameters of underwater fin swimming using dolphin, flutter and breaststroke kicks both with and without diving gear. v_av_−Average swimming velocity; VIV_Index_−Index of variation of intracycle velocity; SI–Stroke index; SL–Stroke length; SR–Stroke rate.

		Swimming without diving gear			Swimming with diving gear		
		Dolphin	Flutter	Breaststroke	Dolphin	Flutter	Breaststroke
	Dolphin		0.000154*	0.000119*		0.119889	0.008223*
**v**_**av**_	Flutter	0.000154*		0.003161*	0.119889		0.124126
	Breaststroke	0.000119*	0.003161*		0.008223*	0.124126	
	Dolphin		0.000119*	0.000059*		0.026548*	0.000126*
**VIV**	Flutter	0.000119*		0.008412*	0.026548*		0.32083
_**Index**_	Breaststroke	0.000059*	0.008412*		0.000126*	0.32083	
	Dolphin		0.000059*	0.000119*		0.217194	0.105785
**SI**	Flutter	0.000059*		0.856860	0.217194		0.624598
	Breaststroke	0.000119*	0.856860		0.105785	0.624598	
	Dolphin		0.000033*	0.008539*		0.005509*	0.000064*
**SL**	Flutter	0.000033*		0.000200*	0.005509*		0.033722*
	Breaststroke	0.008539*	0.000200*		0.000064*	0.033722*	
	Dolphin		0.000059*	0.030384*		0.000059*	0.000033*
**SR**	Flutter	0.000059*		0.000033*	0.000059*		0.022168*
	Breaststroke	0.030384*	0.000033*		0.000033*	0.022168*	

*Statistical significance at p < 0.05.

In the swimming trials without diving gear, all three leg-kicking techniques were significantly differentiated by v_av_ and VIV_Index_, while significant differences between the dolphin kick and the breaststroke kick were found in v_av_ when swimming using diving gear. It should be emphasized that for swimming without diving gear, no significant differences in SI between flutter and breaststroke kicks were found. The same statistical sense was observed in the case of differences between all kicking techniques for swimming with diving gear.

In both trials (swimming with and without diving gear) for all three fin swimming techniques studied, statistically significant differences (Table 1) were found for both stroke parameters (SL and SR) As regards dolphin and breaststroke leg-kicking (both with and without the equipment), significant differences were noted in relation to the “lengthening” of SL and the “reduction” of SR. The same relationship was found for flutter kicking while wearing diving gear. The shortening of SL and increased SR were only observed for flutter kicking without the equipment.

The average swimming velocity (v_av_), stroke index (SI) and the index of variation of intracycle velocity (VIV_Index_) were significantly (Table 1) greater when swimming without diving gear than with it (Fig 4). The values for the stroke parameters SL and SR were also higher when swimming without diving gear. The values of v_av_, SI and VIV_Index_ presented larger differences in trials performed without the diving gear compared to those executed with the equipment. These differences were also noted in flutter and breaststroke kicking for SL and SR.

**Fig 5:**
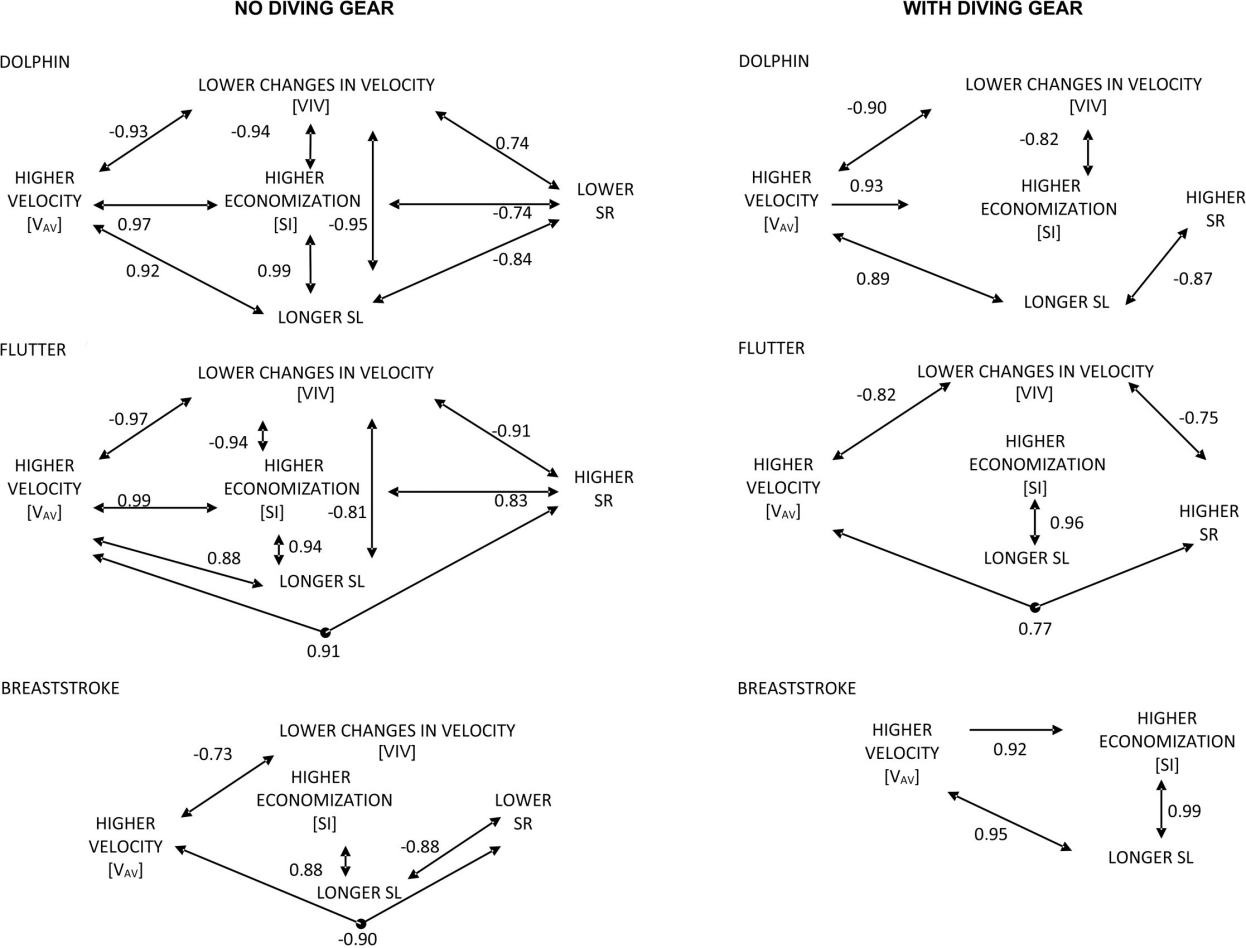
Spearman’s rank correlations between studied parameters, illustrating statistically significant (p<0.05) relations between them. The dependencies between the examined parameters (arrows) exemplify the cause-effect mechanism determining the optimal fin swimming performance while employing different kicking techniques. v_av_−Average swimming velocity; SI–Stroke index; VIV_Index_−Index of variation of intracycle velocity; SL–Stroke length; SR–Stroke rate.

The results of Spearman’s rank correlation between the studied parameters (Fig 5) were analyzed with the assumption that significance of relationships between average swimming velocity (v_av_) and economization of generation (SI) and utilization (VIV_Index_) of kicking propulsion and completeness of them, allow to exemplify the cause-effect mechanisms determining the optimal fin swimming performance while employing different kicking techniques.

In the trials performed without diving gear (Fig 5), an inversely proportional relationship between SL and SR was noted for dolphin kicking compared to flutter kicking, while for the latter in contrast to the former a proportional relationship between SR and v_av_ was observed. For breaststroke kicking, a lack of significant correlations was noted between: v_av_ and SI, SL; VIV_Index_ and SR, SL, SI; SI and SR.

The average swimming velocity (v_av_) of dolphin and flutter kicking without diving gear increase together with the lengthening of SL (positive correlation) and with the minimization of the variation in intracycle velocity—VIV_Index_ (negative correlation), as shown in Fig 5. In the case of dolphin kicking, the lengthening of SL was found to be correlated with reduced SR, whereas in flutter kicking the SL and SR increased together with v_av_. The high average swimming velocity (v_av_) of breaststroke kicking seemed to be the result of a minimization of VIV_Index_. On the other hand, a reduction of the SR in correlation with a lengthening of SL probably indirectly implied a higher value of the SI (in terms of SL to v_av_ ratio).

In the trials performed with diving gear (Fig 5), the high average swimming velocity (v_av_) of dolphin kicking probably resulted from minimizing the SI (SL to v_av_ ratio) minimizing the VIV_Index_, together with lengthening of the SL, associated with reduced SR. For flutter kicking, increased v_av_ went hand in hand with reduced VIV_Index_ and increased SR. The SI and SL did not have an influence on v_av_. In breaststroke kicking the v_av_ increased only as a consequence of proportional relationships between the lengthening of SL and increased SI. It should be emphasized that the set of correlations between parameters relating to swimming with diving gear were suggestively less completed than in trials without the gear.

It was revealed that the cross-sectional areas of the scuba divers’ bodies wearing the equipment were +44.6% larger than when swimming without the gear (Fig 3).

**Fig 6:**
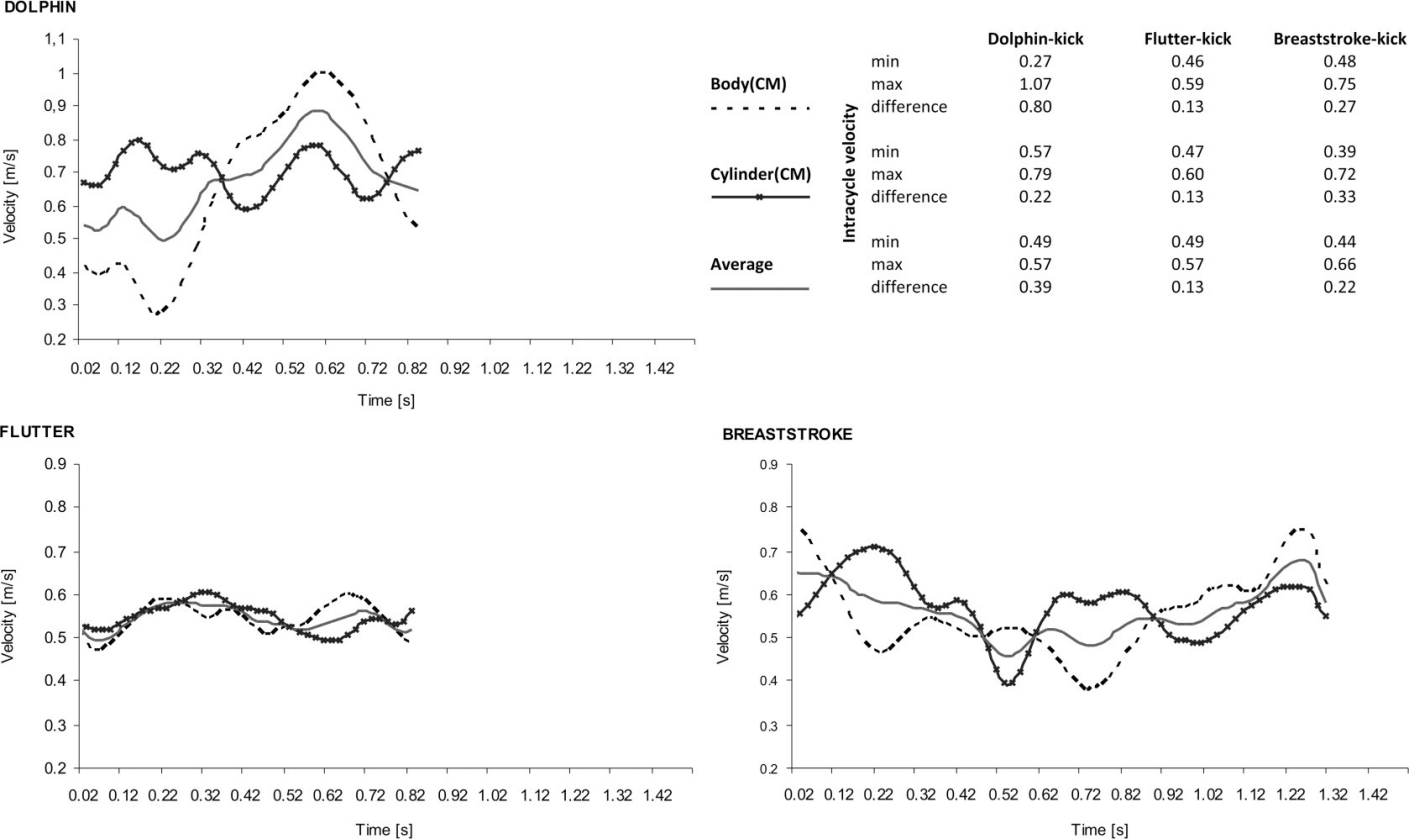
Sample charts of intracycle velocity variations in the diver’s body (CM hip joint), the air tank (CM) and their average series in time function, illustrating the effects of inertia (additional mass of air tank) on the velocity variations of the body-air tank system when swimming with various kicking techniques.

The inertia forces generated as a consequence of displacement of the mass of the air tank, and its influence on the intracycle velocity variations in the swimmer's body and air tank system were also taken into investigation. The sample charts of the intracycle velocity variations of the divers’ bodies (hip joint) and the air tank in time function (Fig 6) showed that the higher the velocity variations, the higher the differences in amplitude between the velocities of the divers and the tank. The highest differences in intracycle velocity were observed in dolphin kick swimming, while the lowest were observed in trials performed with flutter kick. The average series of velocity of the divers’ bodies exhibited the greatest variations when using the dolphin kick and the lowest when using the flutter kick. Moreover, the lowest velocity changes of the air tank were estimated when the divers swam using the flutter kick, and the highest when breaststroke kicking. It is worth noting that when using the flutter kick, the variations in velocities under consideration were in almost the same range of amplitude. For breaststroke kicking the aforementioned ranges were very similar, whereas the maximum and minimum values of velocities clearly differed from each other for dolphin kicking.

It should be noted that the series of intracycle velocity variations of the divers’ bodies in flutter and breaststroke kicking were shifted in phases from the series of velocity variations of the tank (Fig 6). Identical shifting in phases occurred in the first part of the dolphin kick; however, in the second part of the stroke (the downward movement of the legs, leading to maximal intracycle velocity), the aforementioned series were not shifted relative to one another.

## Discussion

The results of a comparison of underwater fin swimming performance using dolphin, flutter and breaststroke kicks both with and without diving gear were interpreted in terms of average swimming velocity, employing the parameters of spatiotemporal structure of the stroke and with reflection to the swimming economy. Intentional controlling of the strategy of propulsion generation in order to achieve optimal performance—oriented on a particular goal of divers was also taken into consideration.

The dolphin kick swimming was the fastest in both trials, (Table 1 and Fig 4). This finding is in accordance with the conclusions reached by Zamparo et al. [14] who studied the fin swimming performance in terms of various velocities. It was also confirmed by the analyses of dolphin kick performed after swimming turns with [29] and without fins [30]. Additionally, the interpretation of the results concerning the swimming velocity in relationship to other parameters (Fig 5), corresponds with the mechanism of propulsion of the fastest fish [31]. The aforementioned references are linked by the general statement that validates the advisability of research in optimization of maximal fin swimming performance [32]. Thus, it seems to be entitled, that the dolphin kick is the best solution for practitioners to enhance fin swimming performance when obtaining a high velocity is the goal of their activity in both, swimming with and without diving gear.

The results did not provide for unequivocal evaluation of the performance in kicking techniques with diving gear. The dolphin kick appeared to be the fastest but not the most economical method of propulsion, although the flutter kick should not be underrated. The results of a comparison of these two techniques can be explained by the results of previous analyses of rescue swimming with fins [2]. When lifeguards approached a victim, the dolphin kick was the fastest technique, but the flutter kick was the fastest when they towed the victim (towing as swimming with additional load can be compared with swimming with air breathing gear). Given the lack of studies pertaining to breaststroke kicking with fins, a justification for the use of this technique needs to be made by analyzing traditional swimming. It is known that breaststroke kick has its own advantages in production of effective/efficient propulsion in underwater swimming when we compare it with swimming performed while breaking the water’s surface. Consequently, a significantly longer stroke length is obtained in comparison to other techniques. Employing the large surface of the fins amplifies this effect, leading to the economization of propulsion utilization (VIV_Index_) when swimming with or without diving gear (Table 1, Fig 4). In this sense, a priori negation of the breaststroke kick as a source of propulsion during underwater fin swimming seems to be unjustified, especially when one realizes how useful the breaststroke kick is in recreational swimming or while performing special tasks (for example, avoiding turbulence in the bottom structure of a water body).

The lowest variations in intracycle velocity (VIV_Index_) while flutter kicking in both trials (Table 1, Figs 4 and 6) are congruent with the results obtained for competitive front crawl swimming [23,33,34]. Significant inverse correlations between VIV_Index_ intracycle velocity variations and average swimming velocity (Fig 5) have also been found for breaststroke [25] and dolphin kicking [35]. According to Barbosa et al. [34] and Vilas-Boas et al. [25], an increase in variations in intracycle velocity caused by increased hydrodynamic drag leads to a reduced swimming performance for all competitive swimming techniques. In this sense, the assumption that VIV_Index_ could reveal of economization of legs' (fins) propulsion utilization has been verified, although this relationship was seen more clearly in swimming without diving equipment.

The stroke index (SI) has been used as a measure of technical skill in all swimming strokes performed in both long and short distances [28]. Zaton et al., [36] presented SI as the tool for assessing economization in three different arm stroke coordinations (“standard”, “looping” and “kayaking”). This parameter positively correlated with the energy cost in front crawl swimming [17]. The results obtained in this study demonstrate that SI did not differ in any of the kicking techniques (Table 1, Fig 4) and correlated with v_av_, VIV_Index_ SR and SL only to a very low extent (Fig 5). Even less correlation was observed when swimming with a diving gear. Thus it could be generalized that SI cannot be considered a reliable diagnostic tool in assessment of underwater fin swimming performance.

A number of studies [10,11,13,21,23,28] revealed that the SR, in relation to the SL, enables swimming performance to be controlled. A strategy for controlling the stroke parameters that determine the performance in fin swimming would help eliminate or minimize the consequences of variations in intracycle swimming velocity [21]. In underwater swimming without diving gear (Fig 5), the fastest dolphin kick technique was characterized by an adjustment of SR in order to maintain the longest SL possible, leading to the most advantageous economy of propulsion generation (SI) and its utilization (VIV_Index_). The flutter kick was not as fast as dolphin kick, probably owing to the increasingly high frequency of leg movements (SR), which directly led to a shortening of the SL. Consequently, in breaststroke kicking, the strategy of low SR when lengthening the SL led to a reduction in the economy of propulsion generation (SI) as well as to the relatively large variations in intracycle velocity, limiting the economization of its utilization (VIV_Index_). Finally, it can be assumed that an optimal strategy for controlling performance in various fin swimming techniques without diving gear comprises lengthening of the SL while keeping the SR at the highest level possible in dolphin and flutter kicking, yet increasing the SR while keeping the SL at the longest level possible in breaststroke kicking.

In underwater swimming with diving gear, a complete layout of the dependencies between the examined parameters was not found (Fig 5). Therefore, only a general proposal for controlling the optimal strategy of swimming performance can be highlighted. These proposals seem to be focused on searching the economical utilization of propulsion (VIV_Index_) through reducing the SR in dolphin kicking, lengthening the SL in flutter kicking and adjusting the SR to keep the SL at the longest level possible in breaststroke kicking. It seems that divers in these trials were unable to control the relationships between SR and SL as precisely as when swimming without diving gear. Therefore, they probably did not meet all necessary conditions to obtain the relatively high performance.

Sanders [37] has stated that besides restraint of physiological cost of effort, the minimisation of negative drag–effecting the human body travelling through water (at any given speed), and maximisation of the positive thrust generated as a consequence of propulsive movements of the swimmer, should be taken into consideration when examining swimming technique in context of the maximisation and/or optimisation of swimming performance. Both depend on the cross-sectional area, the shape and characteristics of the surface, and velocity [38]. This could be seen in the trials performed with diving gear (Table 1, Fig 4), where swimming velocity (v_av_) across the three leg-kicking techniques proved similar despite different relationships between the examined parameters (Fig 5). Indeed, the magnitude of the negative drag that occurs as a result of the large cross-sectional area and the unstreamlined shape of the swimmer wearing the equipment (shown in Fig 3) significantly collapse the possibility of performance.

In search of the causes of inferior performance in underwater swimming with equipment (Table 1, Fig 4), the effects of inertia on the swimmer’s body related to diving gear (air tank) should also be taken into consideration (Fig 6). Colman et al. [39] have shown a positive effect of inertia, explaining that these forces arise as a result of the displacement of added mass of water relative to the swimmer’s body and therefore causing in counterbalancing changes in the intracycle velocity. In this study, this additional mass could be identified as the mass of the air tank. Its displacement probably reduced the changes in intracycle velocity during this part of the breaststroke cycle where swimming velocity decreased (Fig 6). Moreover, it was observed that the higher the mean intracycle velocity variations, the higher the differences between the velocities of the diver’s body and the tank. The biggest similarities between the series of velocities of the body and the tank were observed while flutter kicking and, to a lesser extent, breaststroke kicking. The lowest similarities were observed in trials performed using dolphin kicking. A proper phase shift between the series of velocity variations of the diver’s body and the series of velocity variations of the tank was visible only for dolphin kicking in the first part of the stroke. In the second part (the downward leg movement), the aforementioned series did not shift. It would appear that the inertia induced by the additional mass of the tank while swimming with an excessively high variation in intracycle velocity (high amplitude of undulatory movements and erroneous control of SR and SL) might have negatively affected the performance.

Finally, turning back to consideration of performance in various fin swimming techniques through the prism of praxeology, the thesis can be formulated, that underwater fin swimming performance could be increased through implementation of different kicking techniques as the action-oriented skills in order to reach specific goals.

A small sample size may constitute a potential limitation of this study, however the number of military divers at the highest level of proficiency is a priori limited. Additionally, the nature of their military commitments implies another limitation. Regarding the breaststroke kick preformed while swimming with fins, we would like to point out a high negative impact of this movement structure on the knee function. The swimmers and scuba divers should be aware of possible injuries that might result from external rotation in the knee and hip joints, while applying this technique.

## Conclusions

The motor pattern of dolphin kick seems to be the best solution for diving practitioners to enhance in fin swimming performance when obtaining a high velocity is the goal of their activity in both swimming with and without diving gear.

*A priori* negation of breaststroke kick as a source of propulsion during underwater fin swimming seems to be unjustified, especially when one swims with diving gear. These motor patterns, as is the case of the flutter kick, could be successfully employed to enhance the fin swimming performance when the water activities are more focused on obtaining the utilitarian goals than on fastest swimming.

Given the various goals of underwater swimming, the premises for an optimal strategy of controlling performance should be focused on decreasing the velocity variation, irrespective of the various leg-kicking techniques available. In fin swimming without diving gear, these strategies include the following: lengthening the SL while keeping the SR at the highest level possible in dolphin and flutter kicking and increasing the SR while keeping the SL at the longest level possible in breaststroke kicking. Controlling the relationship between the SR and the SL when swimming underwater with fins and diving equipment may not be as precise as when swimming without them due to the large cross-sectional area of the unstreamlined body as well as large inertial forces induced by the displacement of the air tank relative to the swimmer’s body. Therefore, this strategy could be implemented by decreasing the stroke rate in dolphin kicking, increasing the stroke length in flutter kicking and adjusting the stroke rate in order to keep the stroke length at the longest level possible in breaststroke kicking.
